# Comparison of the Five Danish Regions Regarding Demographic Characteristics, Healthcare Utilization, and Medication Use—A Descriptive Cross-Sectional Study

**DOI:** 10.1371/journal.pone.0140197

**Published:** 2015-10-06

**Authors:** Daniel Pilsgaard Henriksen, Lotte Rasmussen, Morten Rix Hansen, Jesper Hallas, Anton Pottegård

**Affiliations:** 1 Department of Respiratory Medicine, Odense University Hospital DK–5000 Odense C, Denmark; 2 Clinical Pharmacology, Department of Public Health, University of Southern Denmark, Odense, Denmark; 3 Department of Clinical Chemistry and Pharmacology, Odense University Hospital DK–5000 Odense C, Denmark; Örebro University, SWEDEN

## Abstract

**Background:**

While Denmark is well known for its plethora of registers. Many studies are conducted on research databases that only cover parts of Denmark, and regional differences could potentially threaten these studies’ external validity. The aim of this study was to assess sociodemographic and health related homogeneity of the five Danish regions.

**Methods:**

We obtained descriptive data for the five Danish regions, using publicly available data sources: Statbank Denmark, the Danish Ministry of Economic Affairs, and Medstat.dk. These data sources comprise aggregate data from four different nationwide registers: The Danish National Patient Register, The Danish Civil Registration System, The Danish Register of Medicinal Product Statistics, and The Danish National Health Service Register for Primary Care. We compared the Danish regions regarding demographic and socioeconomic characteristics, health care utilization, and use of medication. For each characteristic, one-year prevalence was obtained and analyses were performed for 2013 and 2008 to account for possible change over time.

**Results:**

In 2013, 5,602,628 persons were living in Denmark. The mean age was 40.7 years in the entire Danish population and ranged between 39.6 to 42.4 years in the five regions (coefficient of variation between regions [CV] = 0.028). The proportion of women in Denmark was 50.4% (CV = 0.009). The proportion of residents with low education level was 28.7% (CV = 0.051). The annual number of GP contacts was 7.1 (range: 6.7–7.4, CV = 0.040), and 114 per 1,000 residents were admitted to the hospital (range: 101–131, CV = 0.107). The annual number of persons redeeming a prescription of any medication was 723 per 1,000 residents (range: 718–743, CV = 0.016). Analyses for 2008 showed comparable levels of homogeneity as for 2013.

**Conclusions:**

We found substantial homogeneity between all of the five Danish regions with regard to sociodemographic and health related characteristics. Epidemiologic studies conducted on regional subsets of Danish citizens have a high degree of generalizability.

## Introduction

Studies using administrative data are the backbone of epidemiological research[1]. The Danish population has previously been described as one large cohort[2], and referred to as the epidemiologist’s dream[3]**.** Virtually all medical care in Denmark is furnished by the national health authorities with limited or no co-payment from the patient, and perfect linkage can be achieved using the unique Danish Civil Registration Number assigned to all Danish citizens[4]. The availability of nationwide registries on hospitalizations[5], cancers[6], drug use[7], income[8], and education[9], allows a detailed account of vital health parameters on the level of the single individual. In addition to the nationwide registers, regional registers also exist [10–13]. These registers often have a higher level of detail than the nationwide registers and may allow the researcher to address other research questions. However, this comes at the expense of a smaller sample size, a lower statistical precision and the recurring question of generalizability or external validity, potentially affecting the high quality studies published from these registers [14].

Using publicly available high quality national administrative data, the aim of this study was to assess whether the five regions of Denmark by themselves each can be considered representative samples of the Danish population in terms of sociodemographic parameters and health related utilization and thereby whether studies that employ regional data sources in general should address generalizability as part of their aim.

## Setting

### Overview of the Danish healthcare system

**Fig 1:**
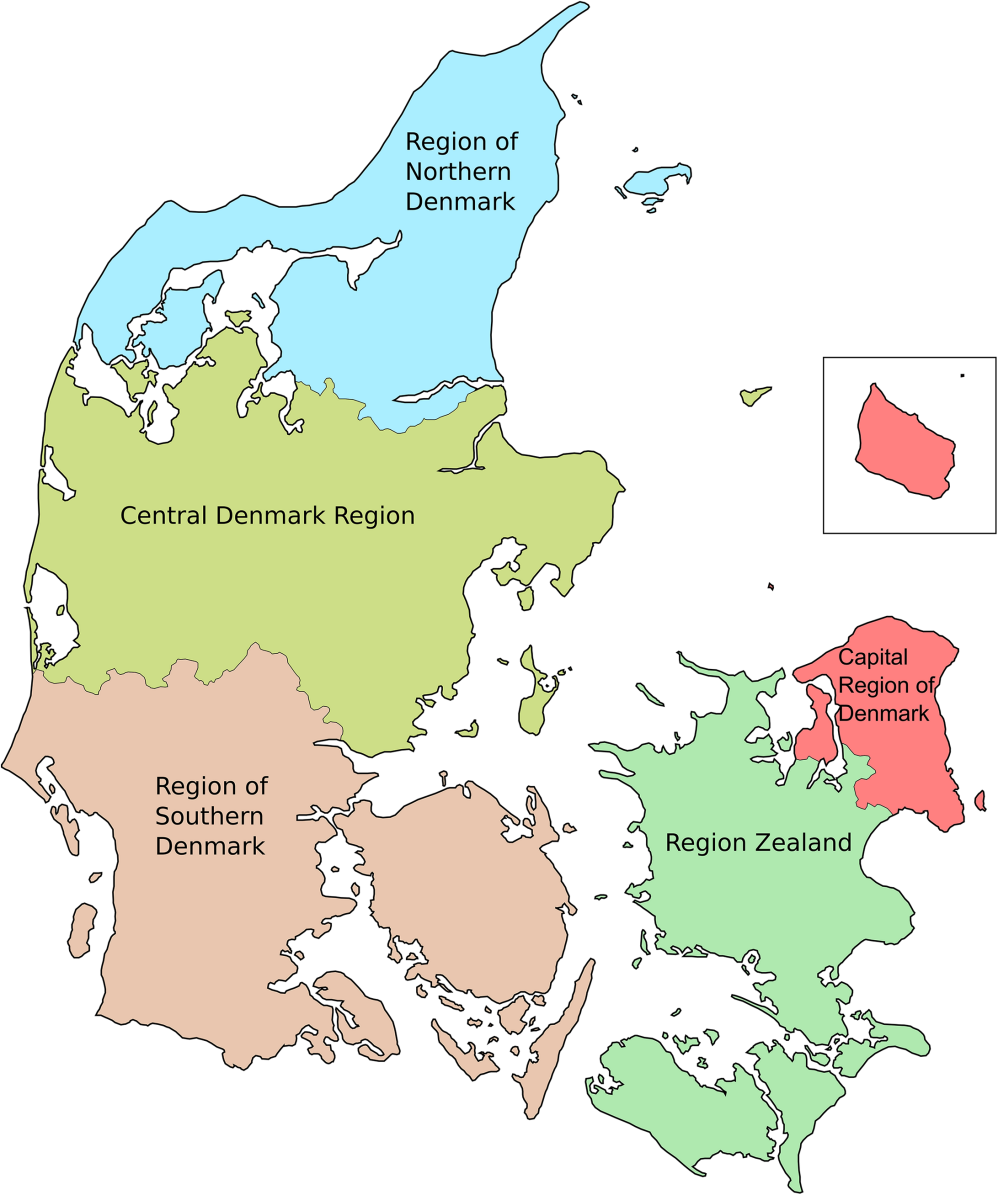
Denmark divided into the five regions: Capital Region of Denmark, Region Zealand, Region of Southern Denmark, Central Denmark Region, Region of Northern Denmark. The figure is licensed under the Creative Commons Attribution-Share Alike 2.5 Generic, 2.0 Generic and 1.0 Generic license. Originally Jarke, modified by Daniel Pilsgaard Henriksen—Derived from http://commons.wikimedia.org/wiki/File:Denmark_regions.svg

Until 2007, the Danish National Health Service was divided into 16 sections: 14 counties and the municipalities Frederiksberg, and Copenhagen, with each county further subdivided into municipalities. After 2007, the counties were combined into five regions[15] (**Fig 1)**.

Each region runs the public hospitals and health services including general practice. A typical general practitioner’s (GP) office receives 95% of its operating income from public funds[16]. Taxes finance approximately 83% of all healthcare expenses, including free access to hospitals, outpatient clinics, GP’s, as well as partial reimbursement of prescribed medicines. The remaining patient copayments make up approximately 17% of the total health expenditures, and primarily constitutes co-payment of medication and dental care[16].

## Methods

### Data sources

We retrieved publicly available aggregate data from four different nationwide registers: The Danish National Patient Register[5], The Danish Civil Registration System[17], The Danish Register of Medicinal Product Statistics[7], and The Danish National Health Service Register for Primary Care[18].

The aggregate data, which was based on data from the four databases above, was retrieved from three different sources:

1. StatBank Denmark is a free-of-charge database containing detailed aggregate statistical information on the Danish Society[19]. The database is hosted by Statistics Denmark, a state institution under the Ministry of Economic Affairs and the Interior[20]. Statistics Denmark was founded in 1850 as an extension of the representative government established by the first constitution of 1849[20]. Statistics Denmark is a governmental institution collecting information electronically provided by administrative registers of different governmental agencies[21]. From this data source, we obtained demographic and socioeconomic data, as well as health utilization (both primary—and secondary care).
2. The Danish Ministry of Economic Affairs and the Interior provides free and public access to key measures of the Danish population[22]. We used this database to identify the proportion of citizens living in urban areas.
3. Medstat.dk contains statistics on the sale of medicines in Denmark based on the data reported to the Register of Medicinal Product Statistics[23]. It is mandatory to report the sale of medicines, and therefore, the data cover all sales from pharmacies and non-pharmacy outlets in Denmark. The sale statistics from primary care are available from 1996 onwards, including a number of individual level parameters. The Register of Medicinal Product Statistics also includes data about medicines sold to hospitals on an aggregate level. Drug use statistics from hospitals are available from 1997 onwards[23].

We used data from 2013 for our primary analysis, but also obtained the same data from 2008 in order to compare the years, and identify potential differences in the population development over time.

### Definitions

#### Demographic characteristics

Age was grouped into 10-year intervals. Population density was defined as number of residents divided by area in square kilometers. Urbanization was defined as the percentage of residents in a region who lived in a town with more than 200 residents. Marital status was categorized as married, never married, divorced, or widowed.

#### Socioeconomic characteristics

Income was reported as the mean income of each person in eight income categories (including individuals ≥15 years of age). Unemployment was defined as the proportion of persons aged 15–64 years unemployed in each region. Education was categorized according to the highest attained educational level: <10 years (primary and lower secondary school), 10–12 years (vocational education and upper secondary school), >12 years (short, medium and long-term higher education)[24].

#### Healthcare utilization

Three measures of healthcare utilization were used: 1) annual number of hospital admissions per 1,000 residents, 2) annual number of outpatient contacts per 1,000 residents, and 3) annual number of GP contacts per resident. GP contacts included daytime visits, telephone consultations, email consultations, and out of hours on-call GPs. A patient could have more than one GP contact per day.

#### Medication use

Two measures of medication use were used: 1) annual number of persons filling a prescription of any medicine per 1,000 residents, and 2) the annual number of persons filling a prescription of the 6 most commonly used main medication categories in the Anatomical Therapeutic Chemical Classification System (ATC) (ATC: A Alimentary tract and metabolism; C Cardiovascular system; D Dermatologicals; J Antiinfectives for systemic use; N Nervous system; R Respiratory system)[25].

### Analysis

We obtained data for the entire Danish population, and stratified by the five regions (Capital Region of Denmark, Region Zealand, Region of Southern Denmark, Central Denmark Region, and Region of Northern Denmark). We presented demographic- and socioeconomic characteristics as 1-year prevalences in absolute numbers and proportions from 2013, and repeated the analyses for the year 2008.

Coefficients of variation (CV) (standard deviations divided by the means) were computed across the five regions as a measure of the differences.

Data management, analyses, and graphics were performed with Stata version 13.1 (Stata Corporation LP ®, Texas, USA).

All Stata codes as well as a detailed guide on how to extract the raw data and reproduce the study results including how to generate the figures are available at http://www.github.com/dphenriksen.

### Ethics statement

The study was conducted entirely based on publicly available aggregate data, and therefore no ethics statement was required.

## Results

### Demographic characteristics

In 2013, 5,602,628 persons were living in Denmark, and 50.4% (N = 2,823,776) were women.

The Capital Region of Denmark comprised 30.9% of the entire Danish population, Region Zealand 14.6%, Region of Southern Denmark 21.4%, Central Denmark Region 22.7%, and Region of Northern Denmark 10.4% (Table 1). The proportion of women in the five regions ranged from 49.7% (Region of Northern Denmark) to 51.0% (Capital Region of Denmark) (Table 1).

The mean age was 40.7 years in the entire Danish population, ranging from 39.6 years (Capital Region of Denmark) to 42.4 (Region Zealand) (CV = 0.028).

**Fig 2:**
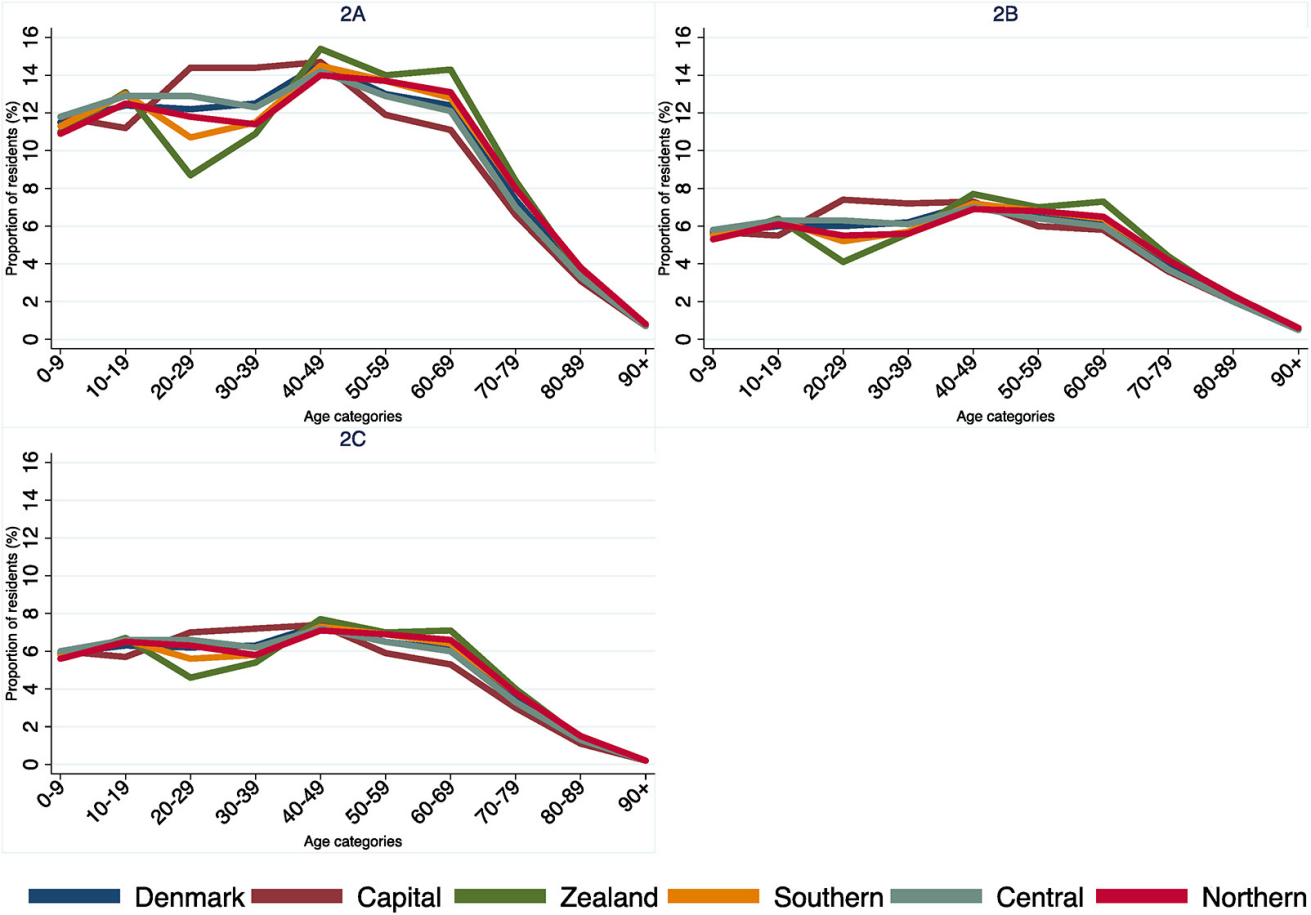
Proportion of residents by 10-year age categories in Denmark, as well as by the five regions in 2013. (2A) Total, (2B) Females, (2C) Males

The proportion of residents in 10-year age categories stratified by gender and region are presented in Fig 2A–2C.

The overall population density was 131 persons per km2 (Table 1). The population density in the five regions ranged between 74 persons per km2 (Region of Northern Denmark) to 679 persons per km2 (Capital Region of Denmark) (CV = 1.230) (Table 1).

**Table 1:** Demographic and socioeconomic characteristics of the Danish population, as well as stratified by the five Danish regions in 2013.

		Denmark	Capital Region of Denmark	Region Zealand	Region of Southern Denmark	Central Denmark Region	Region of Northern Denmark	Coefficient of variation
Total		5,602,628 (100.0%)	1,732,068 (30.9%)	816,359 (14.6%)	1,201,419 (21.4%)	1,272,510 (22.7%)	580,272 (10.4%)	0.395
Gender	Women	2,823,776 (50.4%)	884,093 (51.0%)	411,393 (50.4%)	602,869 (50.2%)	637,052 (50.1%)	288,369 (49.7%)	0.009
	Men	2,778,852 (49.6%)	847,975 (49.0%)	404,966 (49.6%)	598,550 (49.8%)	635,458 (49.9%)	291,903 (50.3%)	0.010
Mean age	Total	40.7	39.6	42.4	41.5	40.0	41.7	0.029
	Women	41.7	40.6	43.4	42.4	40.8	42.7	0.029
	Men	39.8	38.5	41.5	40.6	39.2	40.8	0.031
Population density^a^		131	679	113	98	98	74	1.230
Urbanization^b^	Urban	81%	96%	80%	79%	77%	73%	0.109

^a^ residents per km^2^

^b^ proportion of residents living in cities with ≥ 200 citizens relative to total number of residents in the Region[22].

### Socioeconomic characteristics

**Fig 3:**
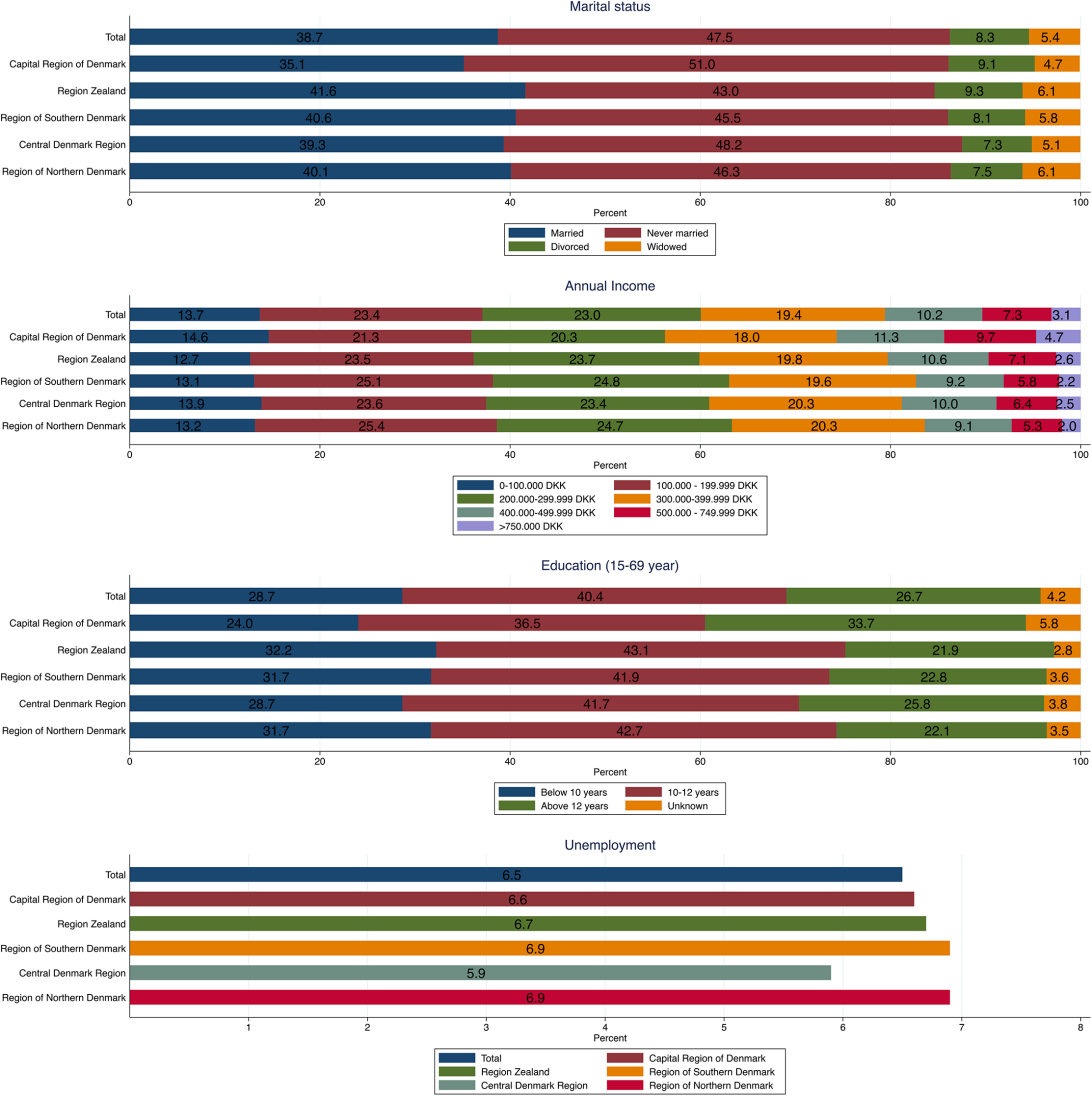
Socioeconomic characteristics (annual income, education, marital status, and unemployment) in proportions of the Danish population, as well as stratified by the five Danish regions in 2013.

Among residents aged 15 to 69 years, a total of 1,140,481 **(**28.7%) had a highest attained education level below 10 years, and 1,062,977 (26.7%) had a highest attained education level above 12 years. The Capital Region of Denmark had the lowest proportion of residents with low education level (24.0%), and Region Zealand had the highest proportion (32.2%). Of all Danish residents, 13.7% were categorized as having an income below 100,000 DKR (below €13,400) ranging from 12.7% (Region Zealand) to 14.6% (Capital Region of Denmark. In total, 3.1% had an income of 750,000 DKR or above (above €100,500) ranging from 2.0% (Region of Northern Denmark) to 4.7% (Capital Region of Denmark) (Fig 3). For results on marital status and unemployment, see Fig 3.

### Healthcare utilization

**Fig 4:**
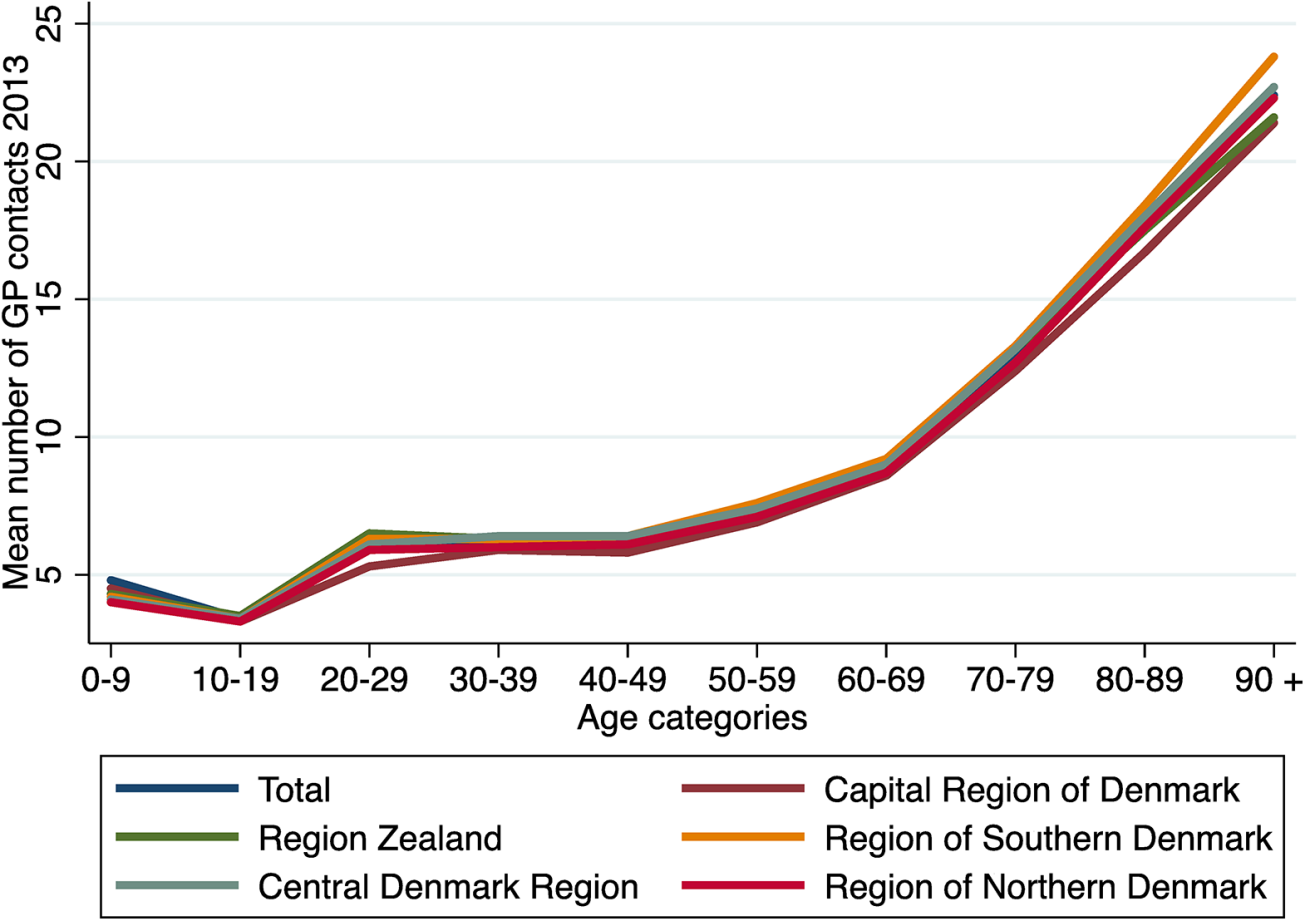
Mean number of contacts per resident to the general practitioners divided into 10-year age categories in 2013.

The annual number of GP contacts for the entire Danish population was 7.1 per resident, and ranged between 6.7 (Capital Region of Denmark) to 7.4 (Region Zealand and Region of Southern Denmark), (CV = 0.041) (Table 2). For the entire Danish population, 114 per 1,000 residents were admitted to the hospital in 2013, ranging from 101 (Central Denmark Region) to 131 (Region Zealand) (CV = 0.107) (Table 2). For a detailed overview of the mean number of GP contacts by age, see Fig 4. For details of outpatient contacts, admissions, and in-hospital bed-days, see Table 2.

**Table 2:** Healthcare utilization of the Danish population, as well as stratified by the five Danish Regions in 2013.

	Denmark	Capital Region of Denmark	Region Zealand	Region of Southern Denmark	Central Denmark Region	Region of Northern Denmark	Coefficient of variation
GP contacts per resident	7.1	6.7	7.4	7.4	7.1	7.1	0.041
Secondary Care utilization (per 1000 residents)							
Outpatients	292	268	283	333	295	282	0.085
Outpatient contacts	1,301	1,302	1,235	1,540	1,203	1,111	0.127
Admitted patients	114	122	131	109	101	107	0.107
Admissions	222	233	284	201	198	199	0.166
Hospital (inpatient) bed-days	721	768	852	671	606	748	0.129

### Medication use

The annual number of persons redeeming a prescription of any medication from a pharmacy was 723 per 1,000 residents in Denmark, ranging from 718 per 1,000 in the Central Denmark Region to 743 in Region Zealand (CV = 0.016). When stratifying on the most frequently used drug classes, we found CV values ranging from 0.026 (ATC: R–Respiratory System) to 0.092 (ATC: C–Cardiovascular system). For details of the prevalences of medication use in the 6 most commonly used ATC main categories, see Table 3.

**Table 3:** Medication use in the Danish population per 1,000 residents, as well as stratified by the five Danish Regions in 2013.

	Denmark	Capital Region of Denmark	Region Zealand	Region of Southern Denmark	Central Denmark Region	Region of Northern Denmark	Coefficient of variation
All medication	723	720	743	739	718	734	0.016
A (Alimentary tract and metabolism)	178	164	193	187	175	194	0.071
C (Cardiovascular system)	244	216	264	265	241	272	0.092
D (Dermatologicals)	179	184	172	180	184	168	0.040
J (Antiinfectives for systemic use)	312	325	326	322	285	301	0.059
N (Nervous system)	235	211	255	251	239	249	0.074
R (Respiratory system)	168	168	173	172	162	168	0.026

### Development over time

Results of demographic-, and socioeconomic characteristics as well as healthcare utilization and medication use from 2008 are presented in S1 File. No differences in heterogeneity were observed in 2008 as compared to 2013. Further, no major differences in the covariate prevalences were found when comparing 2008 to 2013.

## Discussion

In this study, we found that all Danish regions generally can be considered representative of the Danish population in terms of demographic, and socioeconomic characteristics as well as healthcare utilization and medication use. We found that the results were stable over time, when comparing data from 2008 to 2013.

Sample representativeness is important in epidemiological surveys in order to achieve external validity. Population-based samples could, like in the present study, be obtained in terms of a geographical boundary of all persons living within a certain area. Other criteria for sampling include special types of medical contact such as the general practice research databases[26]. In the US, population-based studies are primarily based on persons receiving certain types of social benefits, like Medicare or Medicaid[27], or special groups like the Veterans Affairs Medical Care System[28]. Thus, the large US databases are more limited in scope and underrepresent minority and underserved populations [29].

The large administrative registers like the Danish National Patient Register[5] and the Danish Cancer Registry[6] are all nationwide, and contain information on all Danish residents. However, the regional clinical databases are of special interest, as they often provide additional valuable clinical information like laboratory values[13], redeemed prescriptions from the pharmacies[10,11], or microbiological data[12]. Due to the difficulties in obtaining this additional clinical information on patients, it is therefore important that the source population (the region of interest) resembles the target population (the entire Danish population). Only a single study has addressed this previously[30] but the study only focused on a minor part of Denmark (Northern Jutland and the island of Funen), and much have changed, structurally and politically, since the study was conducted in 1997.

We found that the Capital region had a larger proportion of urbanized area, and a higher population density, than the Danish average, which were the most profound differences we encountered in the data. The Capital region also had a larger proportion of residents with a higher education and a larger proportion of young residents (aged 20–39 years) compared to the Danish average. Other examples of minor differences include a slightly higher rate of outpatient contacts in the South region and a higher rate of admissions in the Zealand region, as well as a lower overall drug consumption in the Central region. In general, however, these differences are only modest, and the residents in the regions appear homogenous in regards to our indicators. This information is of great use to researchers using regional subsets of inhabitants instead of using nationwide samples. Furthermore, it is important to readers of Danish epidemiological research to know that the different regions can be considered a representative sample of the entire Danish population.

There are some limitations associated with the current study. We used only aggregate data, and not data on individual subjects, potentially missing subtle differences within groups. For example, a similar average in hospitalization rate between two regions may coexist with large differences in individual-level distribution of hospitalizations.

## Conclusion

The Danish regions were comparable and can each generally be considered representative of the entire Danish population regarding basic sociodemographic characteristics and health care utilization. Epidemiologic studies conducted on a regional subset of Danish citizens are thus considered to be generalizable to the entire Danish population.

## Supporting Information

S1 File Appendix 1. Results of demographic-, and socioeconomic characteristics as well as healthcare utilization and medication use from 2008
(DOCX)

S2 File Appendix 2. Guide to retrieval of raw data
(DOCX)
