# Supplementary material for: Comparison of the Five Danish Regions Regarding Demographic Characteristics, Healthcare Utilization, and Medication Use—A Descriptive Cross-Sectional Study
Source: PLoS One. 2015 Oct 6;10(10):e0140197. doi: 10.1371/journal.pone.0140197 (PMC4595085; doi:10.1371/journal.pone.0140197)

**Appendix 1**

**Table 1** Demographic and socioeconomic characteristics of the Danish population, as well as stratified by the five Danish regions in 2008.

|  |  | **Denmark** | **Capital Region of Denmark** | **Region Zealand** | **Region of Southern Denmark** | **Central Denmark Region** | **Region of Northern Denmark** | **Coefficient of variation** |
| --- | --- | --- | --- | --- | --- | --- | --- | --- |
| Total |  | 5,475,791 (100.0%) | 1,645,825 (30.1%) | 819,427 (15.0%) | 1,194,659 (21.8%) | 1,237,041 (22.6%) | 578,839 (10.6%) | 0.375 |
| Gender | Women | 2,763,125 (50.5%) | 841,346 (51.1%) | 413,149 (50.4%) | 600,072 (50.2%) | 620,136 (50.1%) | 288,422 (49.8%) | 0.011 |
|  | Men | 2,712,666 (49.5%) | 804,479 (48.9%) | 406,278 (49.6%) | 594,587 (49.8%) | 616,905 (49.9%) | 290,417 (50.2%) | 0.010 |
| Mean age | Total | 40.0 | 39.5 | 41.1 | 40.5 | 39.2 | 40.7 | 0.020 |
|  | Women | 41.0 | 40.7 | 42.0 | 41.5 | 40.1 | 41.8 | 0.019 |
|  | Men | 39.0 | 38.3 | 40.1 | 39.5 | 38.3 | 39.7 | 0.021 |
| Population density, residents per km2 | . | 127 | 643 | 113 | 98 | 94 | 73 | 1.203 |
| Urbanization | . | 80% | 96% | 79% | 78% | 77% | 72% | 0.114 |

^a^ residents per km^2
b^ proportion of residents living in cities with ≥ 200 citizens relative to total number of residents in the Region (source:noegletal.dk)

**Table 2** Healthcare utilization of the Danish population, as well as stratified by the five Danish Regions in 2008.

|  | |  | **Denmark** | | **Capital Region of Denmark** | **Region Zealand** | **Region of Southern Denmark** | **Central Denmark Region** | **Region of Northern Denmark** | **Coefficient of variation** |
| --- | --- | --- | --- | --- | --- | --- | --- | --- | --- | --- |
| **GP contacts per resident** | | | 7.3 | | 7.0 | 7.3 | 7.4 | 7.3 | 7.1 | 0.025 |
| **Secondary Care utilization (per 1000 residents)** | | | |  |  |  |  |  |  |  |
|  | **Outpatients** | | 258 | | 245 | 252 | 282 | 263 | 245 | 0.061 |
|  | **Outpatient contacts** | | 1,060 | | 1,143 | 1,044 | 1,109 | 1,004 | 870 | 0.103 |
|  | **Admitted patients** | | 111 | | 114 | 119 | 109 | 105 | 110 | 0.048 |
|  | **Admissions** | | 198 | | 207 | 218 | 186 | 187 | 191 | 0.071 |
|  | **Hospital (inpatient) bed-days** | | 831 | | 881 | 920 | 768 | 750 | 868 | 0.089 |

**Table 3** Medication use in the Danish population per 1,000 residents, as well as stratified by the five Danish Regions in 2008.

|  | **Denmark** | **Capital Region of Denmark** | **Region Zealand** | **Region of Southern Denmark** | **Central Denmark Region** | **Region of Northern Denmark** | **Coefficient of variation** |
| --- | --- | --- | --- | --- | --- | --- | --- |
| All medication | 736 | 736 | 749 | 751 | 731 | 748 | 0.012 |
| A (Alimentary tract and metabolism) | 162 | 158 | 172 | 167 | 155 | 170 | 0.045 |
| C (Cardiovascular system) | 230 | 212 | 243 | 244 | 223 | 254 | 0.073 |
| D (Dermatologicals) | 184 | 189 | 175 | 184 | 188 | 175 | 0.037 |
| J( Antiinfectives for systemic use) | 332 | 346 | 343 | 342 | 306 | 325 | 0.051 |
| N (Nervous system) | 221 | 206 | 230 | 232 | 220 | 235 | 0.053 |
| R (Respiratory system) | 174 | 176 | 178 | 176 | 168 | 171 | 0.023 |

**Figure 2** - Proportion of residents by 10-year age categories in Denmark, as well as by the five regions in 2008. (2A) Total, (2B) Females, (2C) Males

**Figure 3** Socioeconomic characteristics (annual income, education, marital status, and unemployment) in proportions of the Danish population, as well as stratified by the five Danish regions in 2008.


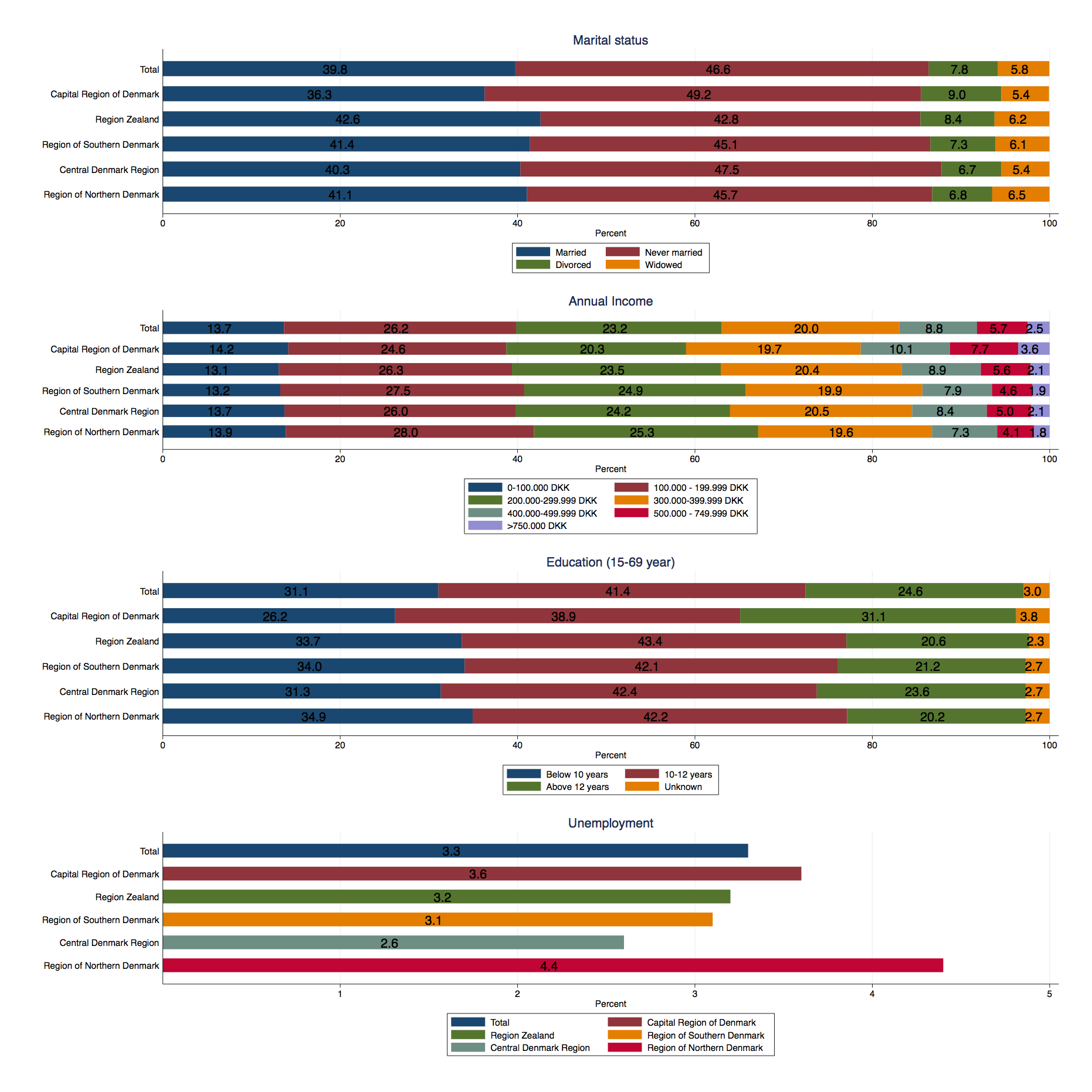


**Figure 4 -** Mean number of contacts per resident to the general practitioners divided into 10-year age categories in 2008. NB a resident could have more than one contact per visit per day.


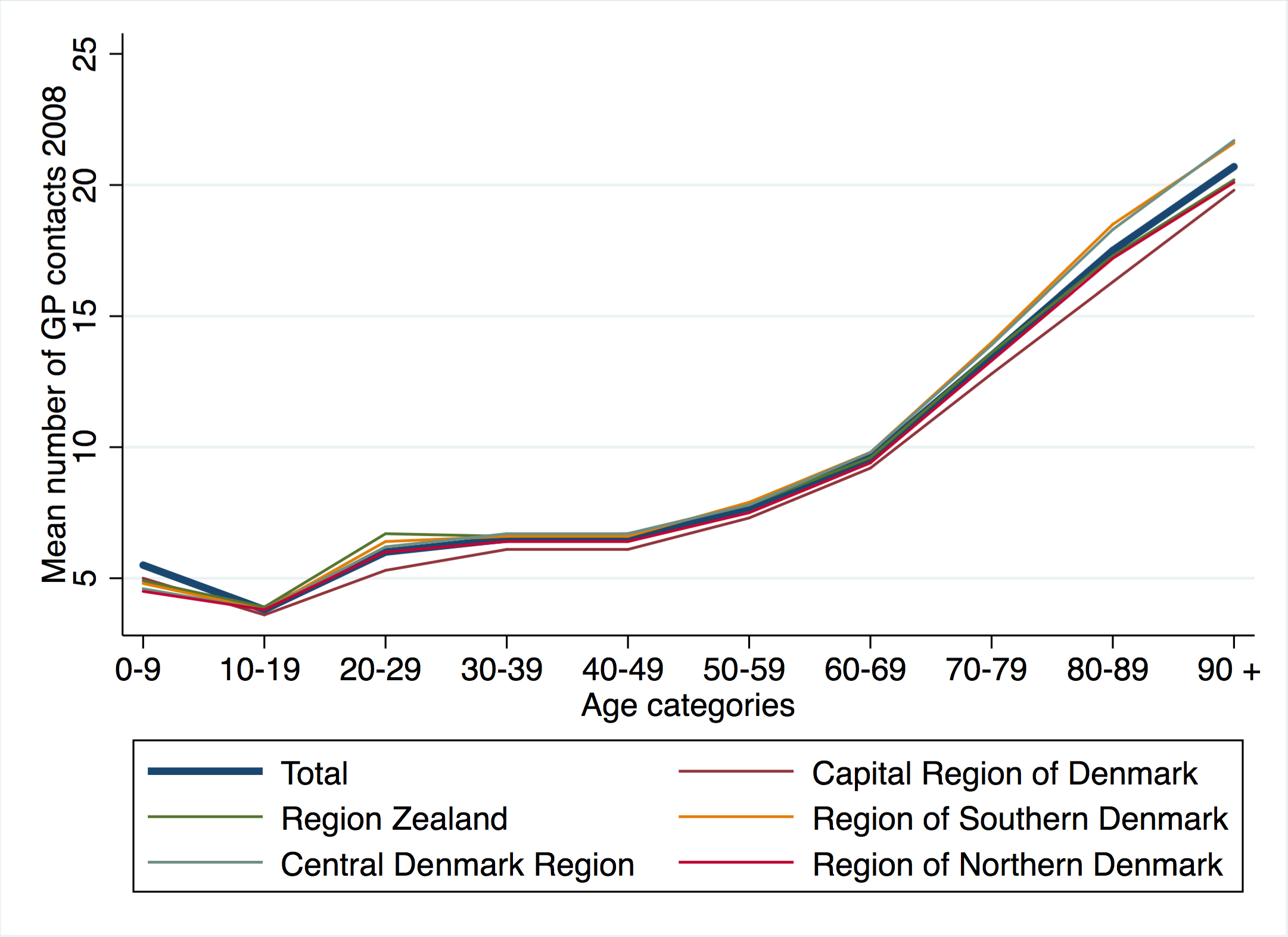

Supplement: S1 File — Results of demographic-, and socioeconomic characteristics as well as healthcare utilization and medication use from 2008 (DOCX) [file pone.0140197.s001.docx]
