# Supplementary material for: Comparison of the Five Danish Regions Regarding Demographic Characteristics, Healthcare Utilization, and Medication Use—A Descriptive Cross-Sectional Study
Source: PLoS One. 2015 Oct 6;10(10):e0140197. doi: 10.1371/journal.pone.0140197 (PMC4595085; doi:10.1371/journal.pone.0140197)
Supplement: S2 File — Guide to retrieval of raw data (DOCX) [file pone.0140197.s002.docx]

**Appendix 2**

**A description of the extraction of data from StatBank, noegletal.dk and medstat.dk.**

**StatBank Denmark (**[**www.statbank.dk**](http://www.statbank.dk) **or** [**www.statistikbanken.dk**](http://www.statistikbanken.dk)**)**

Whenever possible we extracted data from the first quarter from the year concerned (i.e. 2008 and 2013). From StatBank the following variables were used:

The variable **FOLK1** contains data on “population at the first day of the quarter by municipality, sex, age, marital status, ancestry, country of origin and citizenship (2008Q1-2015Q1)”. We used FOLK1 to extract data on marital status, number of residents and the mean age by each region. We collapsed several categories within the group of marital status predefined by the StatBank to obtain our own categorization (e.g. registered partnership was categorized as married). All data were exported from the StatBank into excel files. We renamed the variables afterwards before importing the data into STATA. We used FOLK1 to produce the following data sets: ”1569yr”, ”AgeCat”, ”AgeCat10yr”, ”Gender”, ”Total”, ”Meanage”, “MaritalStatusny”.

The variable **ARE207** contains area by region. We used this variable to obtain the area in square kilometers by each region. The data were exported from the StatBank into one excel file and renamed before importing the data into STATA. We used ARE207 to produce the data set “Area”.

The variable **KRHFU1** contains data on “highest attained education of the population (15-69 years) by region, ancestry, education, age and sex (2006-2014)”. We used KRHFU1 to extract data on the number of residents in each region with different categories of highest attained education. From the education levels in the StatBank we defined the categories used in the study. All data were exported from StatBank into one excel file. We renamed the variables before importing the data into STATA. We used KRHFU1 to produce the data set “Education”.

The variable **INDAMP02** contains data on secondary health care utilization. It contains data on [”population by region, group of diagnosis, key figures, age and sex](http://statbank.dk/statbank5a/SelectVarVal/Define.asp?MainTable=INDAMP02&PLanguage=1&PXSId=0&wsid=cfsearch)”. We only extracted data on secondary health care per 1000 residents. We used the grouping of secondary health care utilization predefined by the StatBank (i.e. out-patients, out-patients treatments, admissions, hospital patients and bed-days). All data were exported from the StatBank into one excel file and renamed before importing the data into STATA. We used INDAMP02 to produce the data set “AdmissionOutPatient”.

The variable **SYGK** contains data on “[contacts covered by the public health insurance by region, type of benefits, age, sex and socioeconomic status](http://statbank.dk/statbank5a/SelectVarVal/Define.asp?MainTable=SYGK&PLanguage=1&PXSId=0&wsid=cfsearch)”. We extracted data on total general medical treatment. All data were exported from the StatBank into one excel file and renamed before importing the data into STATA. We used SYGK to produce the data set “GP”.

The variable **INDKP2** contains data on “income, total by region, unit, sex and income interval”. We used this variable to obtain data number of persons in different income groups from 2008 only. We used the income intervals predefined by the StatBank. We used the variable INDKP102 to obtain the same data from 2013. All data were exported from the StatBank into one excel file and renamed before importing the data into STATA. We used the INDKP2 and INDKP102 to produce the data set “IncomeGroups”.

The variable **AUP02** contains data on “[unemployed in per cent of the labour force (final) by region, age and sex](http://statbank.dk/statbank5a/SelectVarVal/Define.asp?MainTable=AUP02&PLanguage=1&PXSId=0&wsid=cfsearch)”. We used AUP02 to obtain data on unemployment. All data were exported from the StatBank into one excel file and renamed before importing the data into STATA. We used AUP02 to produce the data set “Unemployment”.

**Noelgetal.dk (**[**www.noegletal.dk**](http://www.noegletal.dk)**)**

We used this data source to obtain data on urbanization by each region. First, we extracted data on urbanization by each county, as noelgetal.dk does not allow one to extract data by region. Secondly, we multiplied the measures of urbanization for each county to obtain a total measure of urbanization by each region. By calculating the mean of the measures of urbanization we obtained a value for Denmark in total. We used these data to produce the data set “Urban”.

**Medstat.dk (**[**www.medstat.dk**](http://www.medstat.dk)**)**

We used this data source to obtain data on medication use in each region. We used the ATC code option and marked all relevant ATC codes, years and regions. We only obtained data on sale statistics within the primary health care system and as variable we used number of patients per 1,000 residents. We used these data to produce the data set “Med_use”.
